# The association between reverse total shoulder arthroplasty neck-shaft angle on postoperative patient experienced shoulder disability: a retrospective cohort study

**DOI:** 10.1016/j.jseint.2022.12.013

**Published:** 2022-12-23

**Authors:** Bob Engelen, Esther Janssen, Okke Lambers Heerspink

**Affiliations:** a Department of Orthopaedic Surgery, VieCuri Medical Centre, Venlo, The Netherlands; b Department of Orthopedics and Research School Caphri, Maastricht University Medical Centre+, Maastricht, The Netherlands

**Keywords:** Arthroplasty, Shoulder, Reverse total shoulder arthroplasty, Neck-shaft angle, Patient experienced shoulder disability, Patient-reported outcome measure, Shoulder function, Cuff tear arthropathy

## Abstract

**Background:**

The neck-shaft angle (NSA) of the glenoid component used in reverse total shoulder arthroplasty (RTSA) was reduced to improve functional outcomes. This led to a decreased abduction but increased external rotation ability of patients who underwent RTSA. The impact of the decreased NSA on patient-reported shoulder disability is unknown but may have important implications for functional ability. Therefore, the aim of this study was to assess the difference in patient experienced shoulder disability between an NSA of 135° and 155° 12 months after RTSA.

**Methods:**

In this retrospective cohort study, 109 patients undergoing RTSA were included. In 68 patients, a glenoid component with an NSA of 135° was used and 41 patients received a glenoid component with an NSA of 155°. The primary outcome was Disabilities of the Arm, Shoulder, and Hand (DASH) scores at 12 months and change scores between baseline and 12-month follow-up. Secondary outcomes were complications, Constant Murley Score, Numeric Rating Scale, active forward elevation and external rotation ability. Differences between groups were tested with *t*-tests or Mann-Whitney U-tests.

**Results:**

A mean difference of 10.0 in 12 months postoperative DASH scores between NSA groups was observed in favor of the 135° NSA (*P* = .004), which did not exceed the Minimal Clinically Important Difference. DASH changes scores did not differ between NSA groups (*P* = .652). Mean postoperative Constant Murley Score at 12 months was 11.1 higher in the 135° NSA group (*P* = .013). No differences were observed in complications (*P* = .721) and postoperative pain (*P* = .710) between groups. Difference in postoperative external rotation and forward elevation at 12 months was 10° (*P* = .022) and 20° (*P* = .046), respectively, in favor of the 135° NSA group, exceeding Minimal Clinically Important Differences.

**Conclusions:**

No clinically important difference in patient-reported shoulder disability (DASH) was found between both groups, despite a larger range of motion in the 135° NSA group. This study is the first to show the impact of NSA on patient-reported shoulder disability using the DASH.

Over the last decade, reverse total shoulder arthroplasty (RTSA) has gained popularity as a surgical technique for treatment of cuff tear arthropathy, irreparable proximal humerus fractures, and severe glenohumeral osteoarthritis.^3^^,^^9^^,^^20^

Introduced in 1987, the RTSA design by Grammont proved unique due to its medialized center of rotation. Since its introduction it has undergone several changes, such as inferior baseplate positioning, inferior inclination of the baseplate, and lateralization of the glenosphere, resulting in significantly less scapular notching. To further improve functional outcomes, the neck-shaft angle (NSA) of the glenoid component was decreased.^4^^,^^8^^,^^18^ A recent meta-analysis evaluated the effect of a reduced NSA on shoulder range of motion (ROM) after RTSA. They found decreased abduction but increased external rotation ability in the group of patients with an NSA less than 150°.^10^ Previous studies comparing different NSAs reported improved external rotation and adduction, less scapular notching and fewer dislocations in the 135° NSA group, and no clear association with objective shoulder scores like the Constant Murley Score (CMS), American Shoulder and Elbow Surgeons, or the Simple Shoulder Test.^5^^,^^7^^,^^10^^,^^28^ The decreased abduction ability is accompanied by an increase in adduction ability as per biomechanical studies on 135° NSA.^4^^,^^12^^,^^14^ Theoretically, a trade-off between abduction and adduction by lowering the NSA could offer a functional benefit in daily activities such as toileting.^17^ To the best of our knowledge, no previous study comparing different NSAs in RTSA has reported an impact in patient experienced shoulder disability. Recognizing a possible difference in patient-reported shoulder disability between NSAs could yield important insights for preoperative choice of prosthesis design. Therefore, the objective of this study was to investigate whether a difference in patient experienced shoulder disability could be observed 12 months postoperatively between a 135° NSA and a 155° NSA in patients undergoing RTSA. It is hypothesized that an NSA of 135° leads to a reduction in patient experienced shoulder disability compared to the 155° NSA group.

## Materials and methods

### Study design

Medical ethical approval was obtained for this retrospective cohort study (2021 2899).

### Study population

All adult patients (aged ≥ 18 years) undergoing RTSA between March 2010 and November 2019 in a single centre in Venlo, the Netherlands were included in this study. Patients were included if they underwent RTSA for the following indications: cuff tear arthropathy, glenohumeral osteoarthritis, and revision of hemiarthroplasty or TSA. Exclusion criteria were no available Disabilities of Arm, Shoulder, and Hand questionnaires (DASH) at baseline or 12 months postoperatively, proximal humeral fracture or RTSA revision as indication for RTSA. Exclusion of these indications was performed due to their nondegenerative nature and to prevent duplicate inclusion of shoulders. In this study, two specialized shoulder surgeons performed all RTSA using the deltopectoral approach. Following tenotomy of the subscapularis tendon, glenoid and humeral components were implanted. No reattachment of the subscapularis tendon was performed. The 135° NSA group consisted of patients operated between June 2017 and November 2019 who received a Univers Revers (Arthrex, Naples, FL, USA)prosthesis. In the 135° NSA group, 35 patients received lateralization of the glenospheres by 4 millimeter. No lateralization was performed in the 155° group. The 155° NSA group consisted of patients operated between March 2010 and May 2017 who received an Inverse/Reverse (Zimmer Biomet, Warsaw, IN, USA) or the SMR Reverse (Lima Corp., Villanova, San Daniele del Friuli, Italy) prosthesis.

### Data collection

The primary outcomes were experienced functional shoulder disability at 12 months postoperatively and change scores between baseline and 12-month follow-up, measured using the DASH.^11^ The DASH is a reliable and valid instrument that evaluates multiple dimensions of experienced disability in the arm and shoulder, namely physical function and social/psychological wellbeing. The DASH thereby illustrates a complete patient experience of their shoulder condition on a scale from 0 (minimal disability) to 100 (maximal disability).^2^^,^^29^ Measuring DASH provides an insight into subjective outcomes when compared to an objective shoulder score such as CMS.

The following variables were collected as secondary outcomes: complications, shoulder function measured using the CMS,^16^ shoulder pain measured using the Numeric Rating Scale (NRS)^15^ and active ROM, forward elevation, and external rotation. Complications were reported in the following categories: infection, dislocation, perioperative fissures, acromial fractures, component loosening, scapular notching, nerve lesions, and revision surgery.^1^

Preoperative baseline characteristics collected from electronic patient records consisted of baseline DASH score, sex, weight, Body Mass Index, height, age, previous surgery on ipsilateral shoulder, smoking habit at baseline, diabetes mellitus, cardiovascular comorbidities (cardiovascular event in patient’s history), hypercholesterolemia, operated side, NSA, handedness, American Society of Anesthesiologists classification, Walch classification, and indication for surgery.

### Statistical analysis

The data were analyzed using SPSS (version 26; IBM Corp., Armonk, NY, USA). The significance level was determined at *P* < .050. For the cases in which less than the minimum of 27 questions were answered in the DASH questionnaire, unanswered questions were imputed using stochastic regression imputation to prevent power fall-out. Normality of data was tested using a Shapiro-Wilk test. A *t*-test or its nonparametric equivalent, a Mann-Whitney U test, was performed on DASH scores to test differences in outcomes between the 135° and 155° NSA groups. A Minimal Clinically Important Difference (MCID) of 10.8 for DASH scores was used for determining clinical relevancy.^6^ To correct for possible confounding effects of relevant baseline characteristics that differed between groups, a multivariate linear regression was performed for the association between NSA and 12-month DASH scores. Based on previous research, possible confounders for functional outcomes after RTSA were Body Mass Index, age, and sex.^23^ To adjust for baseline differences between NSA groups, variables with significant baseline differences were included in the multivariate regression analysis. Differences in secondary outcomes between the 135° and 155° NSA groups were compared using a *t*-test or its nonparametric equivalent, a Mann-Whitney U test. MCIDs used for determining a clinical relevant change in secondary outcomes were 10.4 for CMS,^13^ 1.0 for NRS,^19^ 6° for active forward elevation, and 2° for active external rotation.^25^ DASH scores at 12 months were compared within the 135° NSA group between nonlateralized and lateralized patients using a student’s *t*-test. To compare shoulder disability outcomes between surgery indications, a one-way analysis of variance was performed in DASH scores at 12 months.

## Results

**Figure 1:**
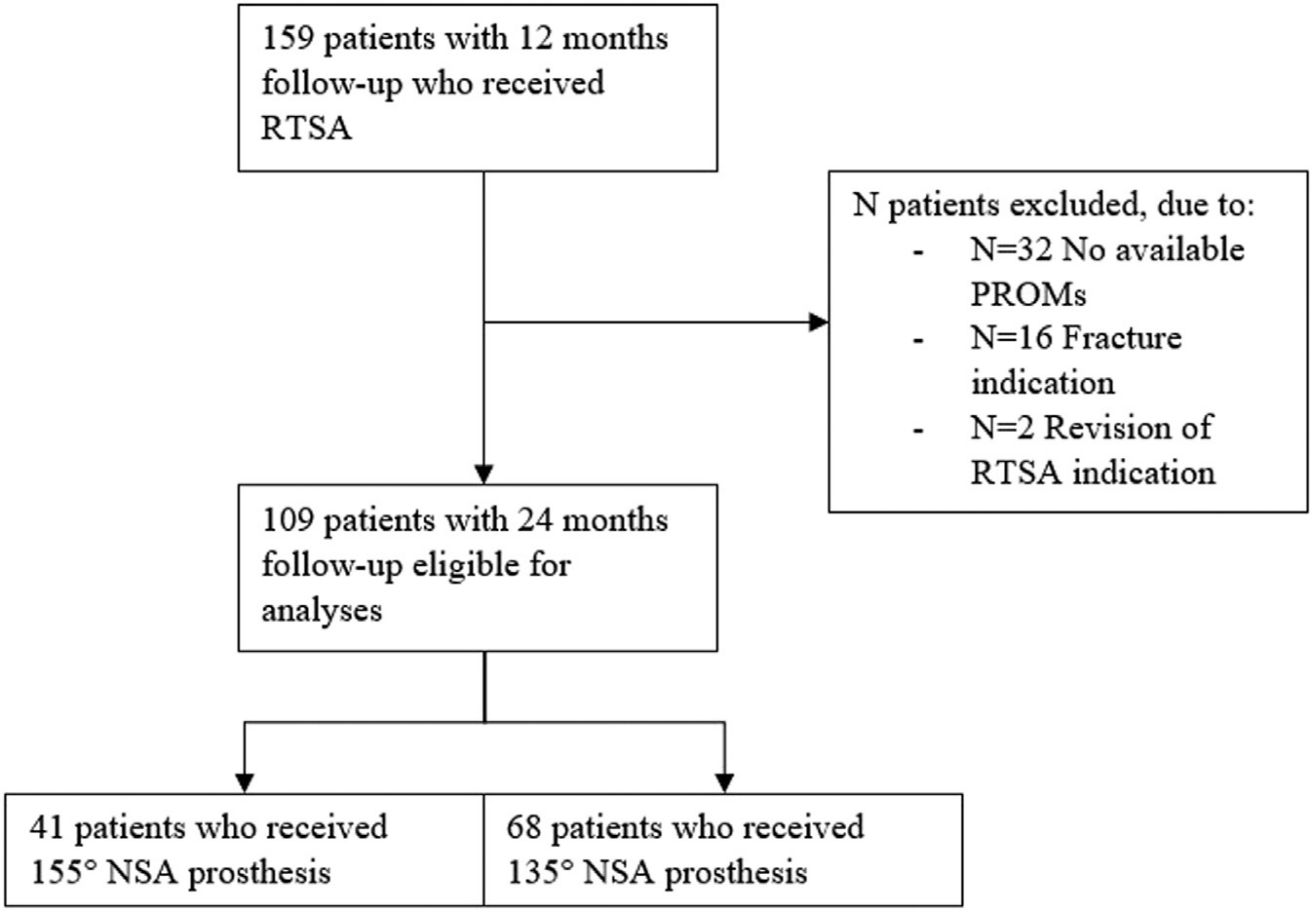
Patient selection. *RTSA*, reverse total shoulder arthroplasty; *PROMs*, patient-reported outcome measures; *NSA*, neck-shaft angle; *N*, number of patients.

In total, 68 patients in the 135° NSA group and 41 patients in the 155° NSA group were eligible for analyses (Fig. 1). Baseline differences were observed between NSA groups in forward elevation ability (*P* = .033, mean 135° = 94.1°, mean 155° = 73.0°, confidence interval [CI]: −40.5°, −1.8°) and DASH scores (*P* = .008, mean difference = 8.1, CI: 2.1, 14.0) (Table I). 23.7% of all DASH answers were imputed. Cuff tear arthropathy was the most common indication for RTSA, occurring in 52.9% of the patients in the 135° NSA group and in 41.5% of the patients in the 155° NSA group. No differences were observed in surgery indications between both NSA groups (Table I).

**Table I:** Baseline patient characteristics

	Total: N = 109	135° N = 68	155° N = 41	*P* value
Sex (N, % female)∗	73 (67.0)	42 (61.8)	31 (75.6)	.137
Weight in kg (mean, ±SD)†	79.6 (15.1)	80.5 (15.4)	78.0 (14.7) (N = 39)	.406
BMI (mean, ±SD)‡	29.5 (5.4) (N = 107)	29.4 (5.30)	29.7 (5.61) (N = 39)	.817
Height in meters (mean, ±SD)†	1.64 (0.1) (N = 107)	1.65 (1.07)	1.62 (0.982) (N = 39)	.137
Age in years at time of surgery, (mean, ±SD)†	74.1 (5.9)	73.8 (5.62)	74.7 (6.43)	.478
Previous shoulder surgery (N, % yes)∗	31 (28.4)	16 (23.5)	15 (36.6)	.143
Smoking (N, % yes)∗	14 (12.8)	11 (16.2)	3 (7.3)	.181
Diabetes (N, % yes)∗	26 (23.9)	12 (17.6)	14 (34.1)	.050
Cardiovascular comorbidities (N, % yes)∗	42 (38.5)	24 (35.3)	18 (43.9)	.371
Hypercholesterolemia (N, % yes)∗	33 (30.3)	25 (36.8)	8 (19.5)	.058
Side (N, % left)∗	35 (32.1)	21 (30.9)	14 (34.1)	.724
Handedness (N, % left)∗	3 (3.2) (N = 93)	2 (2.9)	1 (4.0) (N = 25)	.798
Baseline DASH (mean, ±SD)†	61.4 (15.6) (I = 26)	58.3 (14.4) (I = 14)	66.4 (16.5) (I = 12)	**.008**§
ASA classification (N, %)∗	(N = 108)		(N = 40)	.089
ASA 2	70 (64.8)	40 (58.8)	30 (75.0)	
ASA 3	38 (35.2)	28 (41.2)	10 (25.0)	
Walch classification (N, %)∗	(N = 107)	(N = 67)	(N = 41)	.549
A1	77 (71.3)	46 (67.6)	31 (75.6)	
A2	9 (8.3)	7 (10.4)	2 (4.9)	
B1	6 (5.6)	3 (4.5)	3 (7.3)	
B2	12 (11.1)	9 (13.2)	3 (7.3)	
C	3 (2.8)	2 (3.0)	1 (2.4)	
D	1 (0.9)	-	1 (2.4)	
Indication (N, %)∗				.576
Cuff tear arthropathy	53 (48.6)	36 (52.9)	17 (41.5)	
Omarthrosis	35 (32.1)	21 (30.9)	14 (34.1)	
Post-traumatic	17 (15.6)	9 (13.2)	8 (19.5)	
Other	4 (3.6)	2 (3.0)	2 (4.8)	
ROM				
Forward elevation (mean°, ±SD)†	88.2 (42.2) (N = 89)	94.1 (41.1) (N = 64)	73.0 (41.9) (N = 25)	**.033**§
External rotation (mean°, ±SD)†	25.8 (21.7) (N = 79)	27.7 (22.6) (N = 60)	19.7 (17.8) (N = 19)	.167

*N*, number of patients; *I*, imputated; *BMI*, Body Mass Index; *ASA*, American Society of Anesthesiologists; *ROM*, range of motion; *SD*, standard deviation; *DASH*, disabilities of the arm, shoulder, and hand.

Bold values indicate *P* < .05.

∗ Pearson Chi-squared test.

† student’s *t*-test.

‡ Mann-Whitney U test.

§ *P* < .050.

### Patient-reported shoulder disability

**Figure 2:**
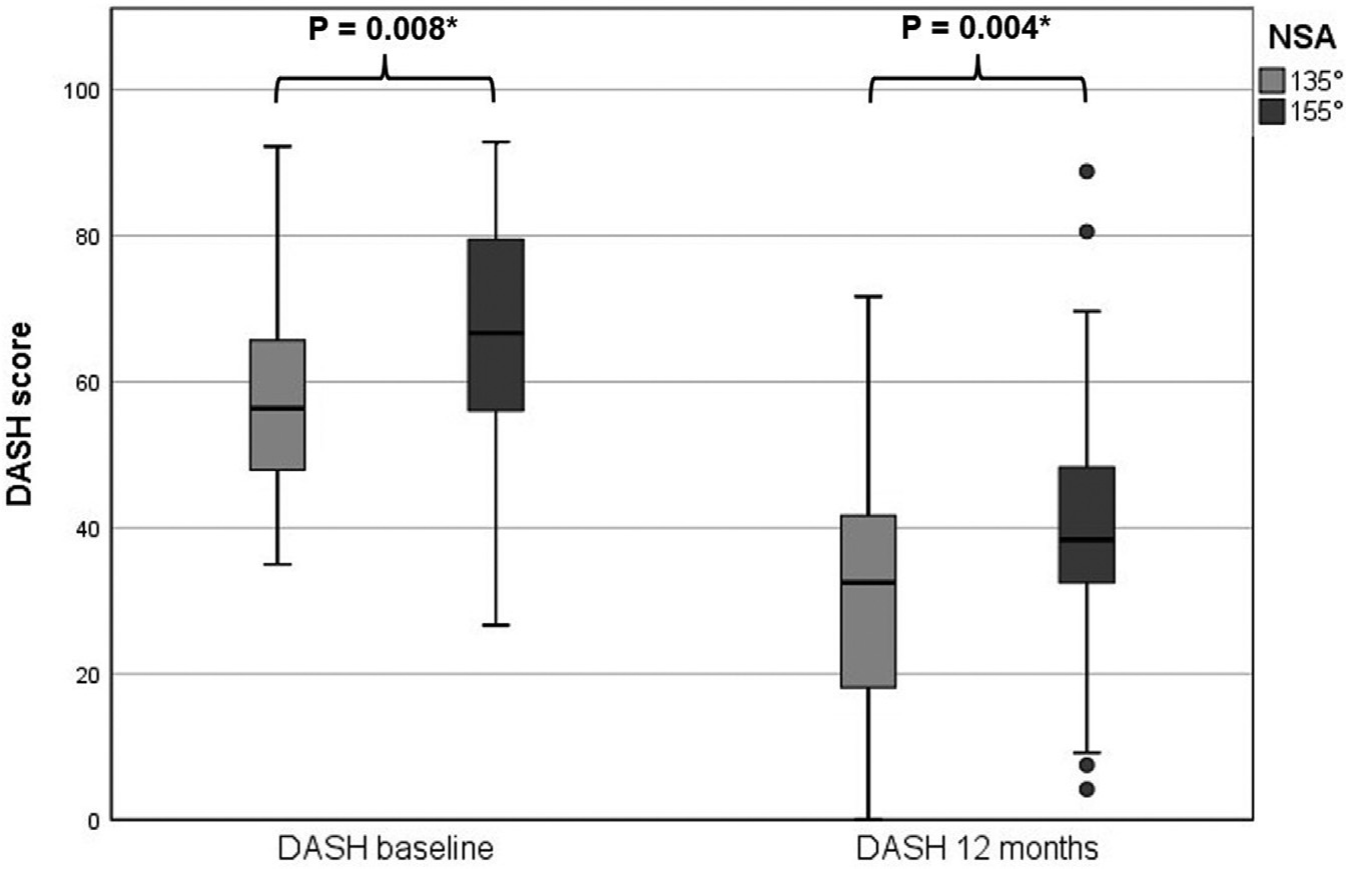
DASH scores at baseline and 12 months between 135° and 155° NSA designs. ∗ = *P* < .05. *DASH*, disabilities of the arm, shoulder and hand; *NSA*, neck-shaft angle.

The mean DASH scores at 12 months were 31.5 (±SD 17.3) in the 135° NSA group and 41.5 (±SD 17.8) in the 155° NSA group (Table II). A difference in DASH scores at 12 months between NSA groups was found, although it did not exceed the MCID of 10.8 (*P* = .004, mean difference = 10.0) (Fig. 2). DASH change scores of −26.9 (±SD 21.5) in the 135° NSA group and −24.9 (±SD 21.6) in the 155° NSA group, both surpassed the MCID. No difference was observed on DASH change scores between NSA groups (*P* = .652, mean difference = 1.9, CI: −6.5, 10.4) (Table II). The difference between NSA groups on DASH scores at 12 months adjusted for confounders remained significant (*P* = .022) (Table III).

**Table II:** Outcome comparison 12 months postoperatively between 135° and 155° NSA groups

	135° N = 68	155° N = 41	*P* value
12 months mean DASH (±SD)∗	31.5 (17.3)	41.5 (17.8)	**.004**†
DASH change score (±SD)‡	−26.9 (21.5)	−24.9 (21.6)	.652
NRS change score (±SD)∗	−4.2 (2.6) (N = 51)	−3.9 (2.7) (N = 17)	.755
12 months mean CMS (±SD)∗	55.5 (12.1) (N = 46)	44.4 (18.8) (N = 27)	**.013**†
12 months mean ROM (°, ±SD)			
Forward elevation∗	126.6 (35.3) (N = 46)	106.7 (31.8) (N = 15)	**.046**†
External rotation∗	32.1 (14.0) (N = 43)	22.5 (13.7) (N = 14)	**.022**†
ROM change scores (°, ±SD)			
Forward elevation‡	28.6 (39.8) (N = 43)	32.7 (49.3) (N = 15)	.750
External rotation∗	3.5 (20.7) (N = 39)	7.1 (11.0) (N = 12)	.352
Complications within 24 months (N, %); subtypes§	13 (19.1)	9 (22.0)	.721
Infection	2 (2.9)	1 (2.4)	.877
Dislocation	2 (2.9)	1 (2.4)	.877
Perioperative fissures	6 (8.8)	1 (2.4)	.188
Component loosening	1 (1.5)	2 (4.9)	.292
Scapular notching	2 (2.9)	4 (9.8)	.131
Nerve lesions	0	1 (2.4)	.196
Revision surgery	2 (2.9)	4 (9.8)	.131

*N*, number of patients; *NRS*, Numeric Rating Scale; *CMS*, Constant Murley Score; *ROM*, range of motion; *SD*, standard deviation; *NSA*, neck-shaft angle; *DASH*, disabilities of the arm, shoulder and hand.

Bold values indicate *P* < .05.

∗ Mann-Whitney U test.

† *P* < .050.

‡ student’s *t*-test.

§ Pearson Chi-squared test.

**Table III:** Confounding correction for the association between 12 month DASH scores and NSA

	Beta coefficient	Confidence interval		*P* value
Constant	70.541	8.865	132.217	.026
Baseline forward elevation	−0.024	−0.114	0.065	.588
BMI	0.194	−0.530	0.917	.596
Age (at time of surgery)	−0.541	−1.234	0.152	.125
NSA	−11.269	−20.837	−1.701	.022
Sex	−4.089	−12.143	3.965	.315
Surgeon	7.463	1.363	13.564	.017
Lateralization	1.537	−7.232	10.306	.728

*BMI*, Body Mass Index; *NSA*, neck-shaft angle; *DASH*, disabilities of the arm, shoulder, and hand.

### Secondary outcomes

Better CMS scores at 12 months were found in the 135° NSA group compared to the 155° NSA group, exceeding the MCID (*P* = .013, mean difference = 11.1, CI: 3.0, 19.3) (Table II). No difference in NRS was observed between NSA groups (*P* = .710, CI: −1.2, 1.7) (Table II). No difference in forward elevation (*P* = .750, CI: −21.4, 29.5) and external rotation (*P* = .352) change scores or complications (*P* = .721) were observed between NSA groups (Table II). A mean difference of 9.6° in postoperative external rotation (*P* = .022) and 19.9° in forward elevation (*P* = .046) at 12 months was found in favor of the 135° group, both exceeding the MCID (Table II).

### Subgroup analysis

In the 135° NSA group, 35 patients received lateralization of the glenospheres by 4 millimeters. Lateralizing the glenosphere showed no difference in DASH scores at 12 months in comparison to nonlateralized cases (*P* = .407, mean difference = 3.5, CI: −11.9, 4.9). One-way analysis of variance showed no differences in DASH scores at 12 months for any surgery indication (*P* = .702).

## Discussion

The aim of this study was to assess the difference in patient experienced shoulder disability at 12 months postoperatively between an NSA of 135° and 155° in RTSA. A significant and clinically relevant difference in CMS, estimated forward elevation, and estimated external rotation was found favoring a 135° NSA. No clinically relevant difference was found between NSA groups in patient experienced shoulder disability. Results from the present study suggest less patient experienced shoulder disability and better ROM in the 135° NSA group after 12 months. However, analysis of change scores showed an equal improvement in both NSA groups on patient-reported shoulder disability and ROM. Both the change scores and 12-month outcomes failed to surpass the clinical relevancy threshold.

Our findings regarding DASH are in line with existing literature by Holsters et al indicating no clinically relevant association between functional shoulder scores and NSA.^10^

Contrary to the DASH, which is a subjective measurement instrument measuring experienced shoulder function, the CMS can be perceived as an objective shoulder measurement instrument of shoulder function, since 65% of the points are given for ROM and strength.^27^ This might explain why clinically relevant differences were found in postoperative CMS results but not in DASH scores. A possible explanation for the difference between subjective and objective outcomes after RTSA is the discrepancy in definition of meaningful improvement from the clinician’s and patient’s perspective. For example, surgeons might interpret an improvement in degrees in forward elevation as a significant improvement, although patients might not be satisfied with their shoulder function until they can perform specific daily activities, which are measured using the DASH. Discrepancy between preoperative expectations of patients and surgeons might also play a role here. A study by Vajapey et al found that high preoperative expectations by patients and their confidence in postoperative improvement were correlated with better postoperative outcomes.^26^ Although this theory might be perceived as counterintuitive, researchers of future studies should be aware of this possible effect and include relevant outcome measures for engaging in meaningful expectation management to ensure optimal patient satisfaction.

Clinical improvement as observed in ROM and objective shoulder measures such as CMS might not represent true patient experienced shoulder disability in an optimal manner. The DASH, being a more subjective patient-reported shoulder measure, is not often used as an outcome measure in RTSA studies, possibly on the basis that DASH is a generalized score for the entire upper extremity.^29^ However, studies by Beaton et al and SooHoo et al showed a good correlation with comparable outcome measures and good validity for use of the DASH in shoulder disorders, justifying its use in future studies aiming to evaluate patient experienced shoulder disability.^2^^,^^22^ Including these outcome measures aimed at eliciting patient perspective provides a more holistic view on a patient’s health status and can therefore better capture the impact of intervention effect on a personal and societal level.

Findings of ROM in this study are partly in line with recent literature. The systematic reviews by Holsters et al and Erickson et al both reported an increased external rotation in 135° NSA when compared to the 155° NSA design but neither found a difference in forward elevation.^5^^,^^10^ Gobezie et al found no difference in forward elevation or external rotation between 135° and 155° NSA designs. Gobezie et al and Erickson et al hypothesized a trend of higher forward elevation in the 155° NSA design.^5^^,^^7^ Our findings suggest a better estimated forward elevation in 135° NSA design and would contradict this hypothesis. Interpretation of the improvement of forward elevation is, as earlier mentioned, not possible as baseline imbalance cannot be ruled out.

### Strengths and limitations

Retrospective cohort studies in particular are limited by data availability and are prone to missing data. In this study, some variables contained missing values and no baseline CMS scores were available. Stochastic regression imputation of the missing data based on incomplete DASH questionnaires was performed to prevent power fallout in the primary outcome variable. This is a reliable method for imputing missing data when auxiliary data are available.^21^ Postoperative missing values in secondary outcomes such as CMS and NRS could possibly have limited the interpretation of these secondary outcomes. The use of different prosthesis systems might result in the misinterpretation of outcomes as an effect of NSA, as in reality, this difference might have been caused by system-specific elements. Systems using a 140° or 145° NSA were not included; therefore, further studies regarding these NSAs are possible. Consequently, the results of this study should not be interpreted singularly but should be confirmed in future larger cohort or randomized controlled trial studies.

### Clinical implications

As orthopedic surgeons, we continuously want to improve outcomes of our treatment. In recent years, an increase in use of a reverse shoulder arthroplasty with a lowered NSA was observed in the Netherlands.^24^ At this moment, mainly short-term follow-up survival data of these newer arthroplasty designs are present. The quality of care must be guaranteed and significant changes to an arthroplasty design should not only bring a significant improvement in functional outcome but also in patient experienced disability to justify the use of a new arthroplasty technique.

## Conclusion

No clinically important difference was observed in experienced shoulder disability between the altered NSA designs despite a better functional outcome for the 135° NSA group. Therefore, we plead to report both objective and more subjective outcome measures in future studies to gain a better insight in which implant design changes are meaningful for patients.

## Disclaimers

Funding: No funding was disclosed by the authors.

Conflicts of interest: The authors, their immediate families, and any research foundation with which they are affiliated have not received any financial payments or other benefits from any commercial entity related to the subject of this article.
